# Miscellany

**Published:** 1902-04

**Authors:** 


### Miscellany.

---

PRECIPITATED SILVER TO HASTEN SETTING OF AMALGAM.—I have found precipitated silver a very effective and convenient agent for hardening amalgam fillings and absorbing the surplus mercury pressed to the surface by the process of packing. Its affinity for mercury is as great as that of freshly annealed crystal gold. Although the crystals are so small that it is practically a powder, it can be applied to the surface of most fillings quite as conveniently as pieces of crystal gold. The most serious objection I know of to its employment is its very low cost. In my own use of it I apply it to fillings in lower teeth generally with tweezers, or a small scoop, and to those in upper teeth by carrying it to place on the point of the finger. None of the silver should be allowed to remain on the surface, as it would be likely to discolor. If the filling is first made more than flush, and cut down and burnished, after hardening, the best results will be obtained.—J. MORGAN HOWE.

---

AN EMERGENCY CROWN.—If necessary, remove the pulp with cocaine. Prepare the root and adjust a post of such length that the lower incisors will not strike it as they articulate, and set it back in the root far enough lingually so that the facing will come into the arch. Flatten slightly the projecting end of the post, and roughen the end to be embedded in the root. Fill the root-canal with a large, soft, gutta-percha cone. Heat the post and grasp the flattened end with suitable pliers, force it to place in the root and trim away the surplus gutta-percha. Slightly roughen the interproximal contact points of the adjacent teeth with a coarse sandpaper disk. Grind the facing to fit properly, roughen the back with a carborundum wheel, and bend down the pins to an angle of forty-five degrees.

Dry thoroughly, mix some of Ames's quick-setting cement, coat the back of the facing, cover the end of the root and post with it, then force the facing to position. Hold until the cement sets slightly, then coat the cutting edges of the lower incisors and have the patient bite; then open the mouth. Trim away the surplus cement, clean out the interproximate spaces, and contour, but leave the cement adhering to the interproximate contact points of the adjacent teeth to give greater security of retention.—J. E. Nyman, *Dental Review.*

---

Celluloid Table-Cover.—I wish to present a little wrinkle that may be of interest to many operators who like neat things and a neat appearance about the chair. The idea is a table-top which can be applied to any swinging table. Secure the correct dimensions, inside measurement, of the top of the table; then obtain from some artists' supply store a piece of sheet celluloid, such as is used for art work, preferably white, and of sufficient thickness to lie flat when put down. It should be fastened in place with thumb-tacks. It is easily kept clean with a dampened cloth, and is noiseless as compared with glass tops, as well as less expensive; yet it has the same advantage that glass has over cloth for this purpose. I have been using a celluloid cover for over a year, and can heartily recommend it to my *confrères.*—Grafton Moore, *Dental Digest.*

---

Facial Neuralgia due to a Hair irritating the Membrana Tympani.—A. Percy Allen, in the *British Medical Journal,* reports the case of a man aged twenty-one years, who had suffered from acute paroxysmal neuralgia for three months.

Having excluded dental and occular causes, the ear on the affected side was examined, and the membrana tympani was seen to be much injected. By means of the auriscope a hair was discovered lying across the meatus, and was pressing with its end against the tympanic membrane. It was removed, and the patient immediately experienced relief. After a day or two the attacks of pain ceased entirely.—*Dental Digest.*

SUGGESTION IN THE ADMINISTRATION OF ANÆSTHETICS.— The *Modern Medical Science* gives the following extract from the *Texas Medical Journal:*

We have made it a rule to employ hypnotic suggestion while administering an anæsthetic, and we are impressed with its value. Our method is to explain to the patient the ease with which one goes under the anæsthetic, and as soon as we begin the administration we say, Breathe deeply, and as you do so, you will find the eyelids getting heavy; you are getting drowsy; you are going to sleep; you are passing into a deep sleep. This method is continued until the patient is seen to be getting very sleepy, when the suggestions are made that you will pass into a deep sleep; that there will be no nervousness or stage excitement; that you will be free from nausea and vomiting, and that when you wake up you will be free from all headaches, nausea, and nervousness. Such a method in our hands seems to have a legitimate use, and we suggest its trial by the profession. Those who are familiar with the usual methods employed in hypnotism are better acquainted with the forms of suggestion indicated and will be most successful in their use.

---

TO PREVENT PAIN WHEN INSERTING THE HYPODERMIC NEEDLE. —The spot where the needle is expected to enter is touched with a toothpick dipped in strong carbolic acid. A white spot immediately appears, due to coagulation of the albumin in the tissues. Shortly after, a perfect anæsthesia of the spot is manifest, and the hypodermic needle can be pushed through the skin or mucous membrane without pain at this point and the infiltration of the tissues begun. If a large arc is to be injected, several spots are marked with the toothpick dipped in the strong carbolic acid, the needle being inserted through these points.— *Medical Standard.*

---

SYNTHOL.—A chemicaly pure substitute for absolute alcohol, known as "synthol," is described in the *Scientific American.* It may be used for every purpose for which alcohol is used except for internal consumption.

Being chemically pure it does not have as much odor as absolute alcohol from grain or wood. It is perfectly free from color, is nonirritant to eyes or skin, and has ten to fifteen per cent. more solvent power than ordinary alcohol. As a killing, fixing, or hardening agent, it is in every respect equal to the best absolute alcohol, and can be used as a substitute for it in the preparation of stains, reagents, etc. As a preservative, it is superior to any alcohol, as alcohol becomes tinged with color on exposure to light, while synthol retains its absolutely colorless condition under all conditions.—*Modern Medical Science.*

---

To PREVENT UNSOLDERING.—In cases where an investment is not indicated it is frequently desirable to observe some precautions to avoid the unsoldering or refusing of parts previously united, which is usually accomplished by the mere presence of the investment itself when such is used.

This may always be very easily prevented by coating or treating such surfaces with crocus (ferric hydrate), or a solution of plumbago, or whiting in water or alcohol.—HART J. GOSLEE, *Items of Interest.*

---

THE USE OF HEAT AS A MEANS OF DIAGNOSING THE PRESENCE OF PUS.—According to Dr. K. Lewin, of Berlin (*British Medical Journal,* January 26, 1901), the application of heat, while relieving pain resulting from simple acute inflammation, is found to have exactly the contrary effect when suppuration is present. Dr. Lewin has applied this observation to the solution of the question of the presence of pus in cases of appendicitis. In ten persons attacked by appendicitis where Dr. Lewin applied hot compresses for one or two hours, eight were greatly relieved, while two found their pains increased.

In all the former group a spontaneous cure resulted in the course of two or three weeks, while in the two cases, after persistent trial of medical treatment without result, operative interference became necessary, and pus was found in both instances.

The author considers that in applying the test it is important to use no other calmative means, and to keep from the patient its meaning, that the effect of the application may not be modified by any dread of an operation.—*Therapeutic Gazette.*

Parr's Fluxed Wax.—This is a hard wax, containing an admixture of borax, which is much used, and is a convenient form of flux, as the wax burns out, leaving the borax deposited upon the surfaces of the metals to be soldered in small proportions.—Hart J. Goslee, *Items of Interest.*

---

Ginger as a Remedy.—Ginger as a remedy is commonly overlooked when a stimulant is needed. It is more popular in domestic practice than with the profession. Its stimulating influence is immediate and greater even than alcohol. It has pain-relieving properties which are difficult to explain. Whenever there is a sudden reduction of the temperature, with coldness of the skin or extremities, or there is chilliness, all accompanying some severe local pain, this agent is a specific.—*Modern Medical Science.*

---

Arsenical Poisoning cured by Orthoform.—I had a case of acute arsenical poisoning with the conditions present so familiar to all. With a spoon excavator I removed the blackened gum-tissue, syringed the parts with warm antiseptic solution, soaked the gum and surroundings with dialyzed iron, and applied twenty-five per cent. unguent orthoform. The pain in this case was the severest that I ever saw, and I must admit being astonished when the patient returned on the following day and reported that the pain had stopped and had not returned. Relief came in twenty minutes.—A. D. Kyner, *Items of Interest.*

